# The Book World of Medicine and Science

**Published:** 1904-06-18

**Authors:** 


### THE BOOK WORLD OF MEDICINE AND SCIENCE.

A MANUAL OF PATHOLOGY. By SIDNEY MARTIN, M.D., F.R.S., F.R.C.P. (London: John Murray. Pp. 502. Price 15s. net.)

THE publication of a manual of pathology by such a well-known authority as Dr. Sidney Martin is assured of a warm welcome and a careful perusal. For many years it has been a standing disgrace that there was no really good English book on pathology and that one had to have recourse to German literature for help. The proof of this statement may be judged from the widespread sale of translations of German pathological works. When it was known that Dr. Martin had produced a new manual, it was hoped that this stigma was now removed. To a certain extent this is so, for the work, as far as it goes, is excellent and superior, we think, to any other book of the kind in the English language. Its fault is that it does not go far enough; in fact its attainment does not reach the height of the author's reputation. The best chapters in the book, in our opinion, are those dealing with infection and immunity. These are well and clearly written, the latest theories on the subject being carefully considered. The chapter on inflammation, due allowance being made for the many difficulties with which the subject is beset, cannot be greeted with an equal approval. In the first place the student is likely to be bewildered by the tabulation of no fewer than nine different varieties of inflammation—a classification at once confusing and unnecessary; and in the second place the article is profusely illustrated by a number of micro-photographs, which for the most part fail to justify their existence. Surely diagrams would serve the purpose better. The chapter on the blood is good, and likewise that on the disorders of the kidney, though here again we have to call attention to the inadequacy of the treatment of the subject. A manual of pathology which only devotes a single page to the complicated question of uræmia cannot be deemed altogether satisfactory. On the whole our feeling towards the work is one of disappointment. There is much good in it and it is a considerable addition to the English pathologicall literature, but it is not sufficiently complete to be accorded unqualified approval.

CONTRIBUTIONS TO PRACTICAL MEDICINE. By Sir JAMES SAWYER, M.D., F.R.C.P. Fourth Edition. (Birmingham: Cornish Brothers. 1904. Pp. 227.)

INSOMNIA: ITS CAUSES AND CURE. [Reprinted from the above.] (Pp. 66.)

SIR JAMES SAWYER has made all members of the profession his debtors by the publication of these contributions to practical medicine. It is pleasing to know, as is indicated by the appearance of a fourth edition, that the book has received a large measure of appreciation. Its substance may be defined as some of the lessons learned in the school of experience by an acute observer engaged for many years in medical practice. With academic doctrines it has small concern. It deals rather with the art of medicine as applied in practical life to the cure of disease and the relief of suffering. The first step to the attainment of these ends is of course accurate diagnosis and this includes an appreciation of the causes as well as a recognition of the existence of abnormal conditions. This essential preliminary to successful treatment is well presented in Sir James Sawyer's chapters, more particularly in those dealing with Insomnia and Gastralgia. The study of these chapters, even apart from their immediate purpose, is an admirable lesson in the method of careful investigation and sound judgment applied to the treatment of individual cases. It is refreshing to read the writings of a physician who, whilst fully competent to expound the pathology of abnormal conditions, recognises the necessity of attempting to devise means for their relief, and who in this attempt can deal with remedies as one familiar with their properties and methods of application. The book is what it professes to be, a contribution to practical medicine, and as such we cordially commend it to our readers.

OPHTHALMOLOGICAL ANATOMY, WITH SOME ILLUSTRATIVE CASES. By J. HERBERT FISHER, M.B., B.Sc.Lond., F.R.C.S.Eng. (London: Hodder and Stoughton. 1904. 8vo. pp. 188. Price 7s. 6d.)

MR. FISHER believes, we think correctly, that "ophthalmic science is to some extent hindered by the want of a clear anatomical picture in the minds of those who devote themselves to it," and he has made a laudable endeavour to supply the requirement to which he calls attention. He describes in successive chapters the visual pathways and centres; the centres for ocular movements; the nuclei, course, and distribution of the cranial nerves; the lenticular ganglion and the cervical sympathetic; the cutaneous nerve areas of the head and face, with the corresponding cerebral topography; the ocular movements and muscles, with an excursus upon squint; the eyelids and the lachrymal apparatus; the ophthalmic blood-vessels and intra-cranial venous sinuses; and the orbit with its surrounding air-cells. In all this there is little or nothing to afford any basis for criticism; and it is only necessary to say that the author has everywhere furnished clear accounts of the relations of the parts which he describes, and that these relations will often elucidate the meaning of symptoms which might otherwise remain obscure. Professed ophthalmologists will be acquainted with the facts, as with parts of the mental furniture necessary for their daily work; but a similar degree of familiarity with them is not to be expected from general practitioners, who will be likely often to find Mr. Fisher's pages useful for the purposes of reference. A few well-chosen if rather rough diagrams will assist in the comprehension of the text.

ASTHMA IN RELATION TO THE NOSE, WITH NOTES OF 402 CASES. By ALEXANDER FRANCIS, M.B., B.C.Cantab. (London: Adlard and Son. 1903. Pp. 136. Price 5s. net.)

THIS volume gives in a more extended form the views which the author has previously published on the nature and treatment of asthma. He regards the nasal apparatus in this condition as morbidly connected with the respiratory centre and thus capable of influencing the asthmatic reflex. The essential part of the treatment of asthma on this hypothesis consists in cauterising the mucous membrane of the nasal septum opposite and immediately above the anterior third of the middle turbinated body. Any nasal abnormality should also be rectified. Complete success is claimed for this treatment in about 60 per cent. of cases, and in only some 5 per cent. is it stated that no benefit resulted, the remainder being greatly improved. The treatment of asthma is in many cases so unsatisfactory that a much smaller proportion of successes would justify a trial of this simple operation.

